# Phytochemical Compounds and Nanoparticles as Phytochemical Delivery Systems for Alzheimer’s Disease Management

**DOI:** 10.3390/molecules27249043

**Published:** 2022-12-19

**Authors:** Saad Bakrim, Sara Aboulaghras, Naoual El Menyiy, Nasreddine El Omari, Hamza Assaggaf, Learn-Han Lee, Domenico Montesano, Monica Gallo, Gokhan Zengin, Yusra AlDhaheri, Abdelhakim Bouyahya

**Affiliations:** 1 Geo-Bio-Environment Engineering and Innovation Laboratory, Molecular Engineering, Biotechnology and Innovation Team, Polydisciplinary Faculty of Taroudant, Ibn Zohr University, Agadir 80000, Morocco; 2 Physiology and Physiopathology Team, Faculty of Sciences, Genomic of Human Pathologies Research, Mohammed V University in Rabat, Rabat 10100, Morocco; 3 Laboratory of Pharmacology, National Agency of Medicinal and Aromatic Plants, Taounate 34025, Morocco; 4 Laboratory of Histology, Embryology and Cytogenetic, Faculty of Medicine and Pharmacy, Mohammed V University in Rabat, Rabat 10100, Morocco; 5 Department of Laboratory Medicine, Faculty of Applied Medical Sciences, Umm Al-Qura University, Makkah 21955, Saudi Arabia; 6 Novel Bacteria and Drug Discovery Research Group (NBDD), Microbiome and Bioresource Research Strength (MBRS), Jeffrey Cheah School of Medicine and Health Sciences, Monash University Malaysia, Subang Jaya 47500, Malaysia; 7 Department of Pharmacy, University of Naples Federico II, Via D. Montesano 49, 80131 Naples, Italy; 8 Department of Molecular Medicine and Medical Biotechnology, University of Naples Federico II, Via Pansini 5, 80131 Naples, Italy; 9 Department of Biology, Science Faculty, Selcuk University, 42130 Konya, Turkey; 10 Department of Biology, College of Science, United Arab Emirates University, Al Ain 15551, United Arab Emirates; 11 Laboratory of Human Pathologies Biology, Department of Biology, Faculty of Sciences, Mohammed V University in Rabat, Rabat 10106, Morocco

**Keywords:** Alzheimer’s disease, neuroinflammation, bioactive compound, nano-drugs

## Abstract

Alzheimer’s disease remains one of the most widespread neurodegenerative reasons for dementia worldwide and is associated with considerable mortality and morbidity. Therefore, it has been considered a priority for research. Indeed, several risk factors are involved in the complexity of the therapeutic ways of this pathology, including age, traumatic brain injury, genetics, exposure to aluminum, infections, diabetes, vascular diseases, hypertension, dyslipidemia, and obesity. The pathophysiology of Alzheimer’s disease is mostly associated with hyperphosphorylated protein in the neuronal cytoplasm and extracellular plaques of the insoluble β-amyloid peptide. Therefore, the management of this pathology needs the screening of drugs targeting different pathological levels, such as acetylcholinesterase (AchE), amyloid *β* formation, and lipoxygenase inhibitors. Among the pharmacological strategies used for the management of Alzheimer’s disease, natural drugs are considered a promising therapeutic strategy. Indeed, bioactive compounds isolated from different natural sources exhibit important anti-Alzheimer effects by their effectiveness in promoting neuroplasticity and protecting against neurodegeneration as well as neuroinflammation and oxidative stress in the brain. These effects involve different sub-cellular, cellular, and/or molecular mechanisms, such as the inhibition of acetylcholinesterase (AchE), the modulation of signaling pathways, and the inhibition of oxidative stress. Moreover, some nanoparticles were recently used as phytochemical delivery systems to improve the effects of phytochemical compounds against Alzheimer’s disease. Therefore, the present work aims to provide a comprehensive overview of the key advances concerning nano-drug delivery applications of phytochemicals for Alzheimer’s disease management.

## 1. Introduction

Alzheimer’s disease (AD) is a neurodegenerative disease that constitutes a critical public health and medical practice issue for populations around the world. It is a complex and challenging neurological condition that is typically characterized by impairments in cognitive and/or motor abilities as well as progressive loss of episodic memory, frequently associated with behavioral abnormalities such as apathy, anxiety, irritability, depression, and aggression [1]. The onset of this illness also has a considerable impact on the family life of the patient, in addition to a significant economic burden for society [2]. This challenging condition is attributed to a combination of several risk factors, some of which we cannot control, such as our age and genetics, as well as others that we can do by taking measures to ensure our brain health. It has been established that approximately 70% of the risk of developing AD can be ascribed to genetic factors. On the other hand, some acquired factors such as hypertension, stress, smoking, marital status, inadequate sleep, depression, cerebrovascular disease, dyslipidemia, obesity, and diabetes significantly increase the risk of developing this disease [1]. Although extensive research has been conducted, the exact mechanisms that activate AD are still not known [3]. However, from a pathophysiological molecular aspect, the occurrence of extracellular plaques of insoluble β-amyloid peptide (Aβ) and neurofibrillary tangles (NFTs) with hyperphosphorylated tau protein (P-tau) in the neuronal cytoplasm constitute the distinctive feature of AD [4]. Whereas it is still debated by what mechanisms these changes lead to cognitive decline, it is suggested that these deposits are responsible for the death and neuronal atrophy resulting from the processes of excitotoxicity, inflammation, depletion of neuronal and energetic factors, and collapse of calcium homeostasis. Following this event, neuronal and synaptic damage implicated in the processes of memory, and learning and other cognitive abilities cause the cognitive decline mentioned above [5]. It should also be noted that in recent years, despite the investigation and evaluation of multiple complex signaling pathways related to AD neuropathology, such as glycogen synthase kinase-3β (GSK3-β) and the study of the interaction between multiple pathological and risk factors, the discovery of very potent therapies with multifaceted curative properties seems necessary because to date there is no treatment for this disease [3]. For this reason, the use of phytochemical research seems to be a promising alternative to finding solutions and developing new drugs against this complex neurodegenerative disease. Dietary bioactive molecules such as polyphenols, sterols, flavonoids, alkaloids, vitamins, lignans, triterpenes, and tannins have been shown to form the main part of traditional prescriptions and have been the subject of extensive research for their inclusion in the exploration of contemporary medicine [6,7,8,9,10,11].

Moreover, previous studies have reported that these natural bioactive molecules exhibit anti-inflammatory, anti-amyloidogenic, and antioxidant activities and target autophagy; therefore, they may be able to offer a very high neuroprotective potential and mitigate the progression of AD lesions [12,13]. Nevertheless, the majority of these candidates possess relatively low degrees of bioavailability, stability, solubility, and target specificity in the body, rendering the presence of these compounds at their effective levels in the target tissues impractical [14]. In this regard, the nanoformulation of these components into nanoparticles provides a new opportunity to overcome the above challenges. Phytochemicals can be encapsulated in biodegradable, biocompatible nanoparticles to boost their bioavailability, aqueous solubility, and stability [15]. Polymeric nanoparticles, nanoliposomes, and nanoemulsions are the most widely used examples of nanoparticles for drug delivery and the biodistribution of natural products, especially phytochemicals [16]. In this review, we will first provide an updated overview of AD, and secondly, we will discuss profoundly natural bioactive compounds with biological properties targeting different signaling pathways involved in the development of AD and their nano-formulation for the management of this neurodegenerative disease.

## 2. Alzheimer’s Disease

### 2.1. Risk Factors and Pathophysiology

AD is not a single illness, according to the nomenclature. There is evidence that missense mutations in the presenilin 1 or 2 genes on chromosomes 14 and 1, or the APP gene on chromosome 21, cause early-onset autosomal dominant familial AD [17]. At the same time, the bulk (90–95%) is of sporadic origin. Cellular/molecular risk factors for this illness are thought to be age-related changes in membrane composition and glucose/energy metabolism, as well as sympathetic tone in the brain [18].

Indeed, synapses are the first to show pathological signs in AD before the onset of clinical symptoms. Due to the high energy demands of synaptic activity, interruption of mitochondrial energy supply may be a primary component in synaptic failure in AD. This function may be compromised by a recently reported age-related reduction in neuronal NADH and redox ratio [19].

In AD, however, glucose uptake and metabolism in the brain are altered (AD). The levels of the two main cerebral glucose transporters (GLUT1 and GLUT3) responsible for glucose uptake in neurons are reduced in the brains of AD patients, according to Et al. [20]. This decline coincides with a decrease in O-GlcNAcylation, tau hyperphosphorylation, and neurofibrillary tangle density in the human brain [20].

The early or facial type of AD is caused by the dominant genetic impact of mutations in amyloid precursor protein (APP), presenilin-1 (PS1), or PS2.

The “amyloid cascade” hypothesis in AD pathogenesis has been shown to dramatically alter APP metabolism, boosting the formation of aggregation-prone Aβ species [21]. This generally recognized concept suggests that the generation of neurotoxic Aβ peptides by β-secretase and γ-secretase is the origin of AD pathophysiology, with all other disease characteristics evolving as a result of this event [21].

Although the amyloid cascade theory is likely to apply to the familial type of the illness, a growing body of evidence shows that the processes causing late-onset AD—the vast majority of AD cases—may be different [22].

Undoubtedly, the best-studied proteins related to AD pathogenesis are amyloid and microtubule-associated tau protein. It is not unexpected that the pathological diagnosis of AD is based on both amyloid and protein deposits [23,24].

Under physiological conditions, tau is a microtubule-associated protein that is sorted into neuronal axons. Tau sorting processes fail in AD and other tauopathies, and Tau is mis-sorted into the somatodendritic compartment. In AD, abnormal amyloid (Ab) synthesis can lead to Tau mis-sorting [25]. Another characteristic of AD is granulo-vacuolar degeneration in the pyramidal cells of the hippocampus. This degradation is most likely connected with cognitive decline; changes in cognitive processes coincide with a decrease in presynaptic bouton density in pyramidal neurons of lamina III and IV [26].

A better method would be to examine AD as an event associated with changes impacting entire biological networks. A variety of processes have been implicated, including neuroinflammation [27], oxidative stress [28,29], impairments in mitochondrial dynamics and function [30], cholesterol and fatty acid metabolism, and changes in brain glucose energy pathways [31,32]. Cell cycle alterations and oxidative stress caused by increased ROS (reactive oxygen species) and RNS (reactive nitrogen species) have also been proven to be deleterious in AD [33,34]. Not all cases of AD are sporadic; a tiny percentage are hereditary.

Mitochondrial malfunction may have a significant impact on early AD. Damaged mitochondria are less effective ATP generators and more efficient ROS producers; therefore, it is likely not a coincidence that lower energy generation, increased oxidative stress, and damaged mitochondria are all characteristics of AD [35,36].

### 2.2. Symptoms: Features and Management

AD is a neurological illness that causes progressive deterioration of behavioral and cognitive abilities such as memory, comprehension, language, attention, thinking, and judgment.

The most frequent first symptom is episodic short-term memory loss with significant sparing of long-term memory, which may be caused in most individuals even if it is not the primary symptom [37].

Apathy, anxiety, and irritability are the first neuropsychiatric symptoms associated with AD. In the latter stages of dementia, disturbances in appetite and sleep, disinhibition, and changes in perception (hallucinations) or thinking (delusions) are common. In addition to conventional neuropsychiatric symptoms, anosognosia (i.e., loss of insight) frequently occurs early and presents a serious treatment dilemma [38].

Furthermore, evidence suggests that a long “dormant period” of progressive oxidative damage precedes and leads to the apparent sudden onset of clinical and pathological symptoms of AD, such as amyloid β deposition, neurofibrillary tangle formation, metabolic dysfunction, and cognitive decline [39]. Early-onset AD has more hippocampal sparing and posterior neocortical atrophy, more tau burden, and more connectomic alterations impacting frontoparietal networks rather than the default mode network [40]. Indeed, the diagnosis of AD is based on a clinical-neuropathological assessment. The gold standard for diagnosis remains neuropathological evidence of β-amyloid plaques, intraneuronal neurofibrillary tangles (including tau protein), and amyloid angiopathy [41].

### 2.3. Management of Alzheimer’s Disease

Cholinergic impairments are the most common neurochemical alterations in AD patients. Several pharmaceutical treatments have been attempted to remedy these deficiencies. Among these, acetylcholinesterase inhibition is currently the most effective treatment for AD.

The Food and Drug Administration (FDA) has authorized AchE inhibitors tacrine, donepezil, rivastigmine, and galantamine [42]. Donepezil is the most widely used AD medication in more than 50 countries. Donepezil is a highly selective and reversible piperidine derivative with AchEl activity that has the best pharmacological profile in terms of cognitive improvement, responder rates (40–58%), discontinuation (5–13%), and side effects (6–13%) in AD [43]. Donepezil has been demonstrated to improve patients’ cognitive and general functioning. However, due to a variety of side effects, more individuals receive high dosages of donepezil-terminated therapy [44].

Donepezil, on the other hand, has been demonstrated to be beneficial and safe in Chinese AD patients and may influence AD biomarkers such as hippocampal atrophy, Aβ, and tau. Furthermore, apolipoprotein E or cytochrome P450 2D6 polymorphism may alter the donepezil treatment response [45].

Long-term administration of memantine to patients with AD improved overall clinical symptoms, cognitive function, and SCPD, thereby reducing caregiver burden and transiently inhibiting reduced cerebral blood flow in the prefrontal area [46].

Both as monotherapy and in combination with donepezil, memantine improves cognitive functions and behavioral disorders more effectively than a placebo.

Despite the fact that memantine monotherapy and combination treatment are linked with specific individual side effects such as drowsiness, memantine is well tolerated, and its safety (all-cause discontinuation) is equivalent to or better than placebo (agitation). According to Matsunaga et al., the combination of memantine with donepezil is the most effective treatment for AD [47].

Tacrine, the first cholinesterase inhibitor licensed for the treatment of AD, could not arrest the course of the disease and showed only minor cognitive gains, but it was associated with serious adverse effects, including hepatotoxicity. As a result, the medication was pulled from clinical trials. Numerous articles have described non-hepatotoxic tacrins since then, and substantial attempts have been undertaken to create multi-targeted tacrins by combining their cholinesterase inhibition profile with the regulation of other biological targets involved in AD [48]. Rivastigmine is a slowly reversible dual inhibitor of acetyl and butyryl cholinesterase that is selective for the G1 isoform of acetylcholinesterase and does not undergo hepatic metabolism by the CYP450 system. Despite its distinct qualities, it has been linked to a greater rate of adverse events when compared to other ACE inhibitors. The oral form, which is approved for the treatment of mild to moderate AD, is associated with a higher rate of gastrointestinal side effects [49].

Rivastigmine (or Exelon) is a cholinesterase inhibitor that is now used to treat the symptoms of mild to moderate AD. Endoproteolytic β-secretase (or BACE1) and y-secretase produce Aβ peptide from its precursor protein (APP). Alternative cleavage of APP by α-secretase (a family of membrane-bound metalloproteases-Adamalysins) results in a neuroprotective and neurotrophic released sAPPα fragment [50].

### 2.4. Pharmacological and Non-Pharmacological Strategies

Only symptomatic therapies are currently available for this condition, all of which aim to prevent neurotransmitter disruption; three cholinesterase inhibitors and memantine. To slow disease development, treatment drugs are thought to interfere with the pathogenic stages that cause clinical symptoms, such as the creation of extracellular-amyloid plaques and intracellular neurofibrillary tangles [51]. Non-pharmacological treatments are classified into four types: holistic approaches, short psychotherapies, cognitive methods, and alternative tactics. Reality orientation and cognitive stimulation therapy (CST) have been associated with considerable improvements in cognition and behavior in people with mild to moderate AD, as well as with reinforcement of concomitant pharmaceutical treatment [52].

Electrical or magnetic brain stimulation can cause neuroplastic changes, and so has therapeutic promise. Long-term effects on cerebral glucose metabolism, a sign of synaptic activity, have been reported when the therapy is repeated [53]. Indeed, studies have shown a transitory improvement in a face-name association memory test in senior participants [54], as well as in object and action naming, even in advanced stages of AD [55]. Increased brain plasticity might be part of the process underpinning cognitive benefits linked with cognitive treatments [56]. Cognitive training tries to maintain or develop a specific element of cognitive functioning (e.g., memory or attention) through systematic, guided practice performed alone or in groups [57]. The level of difficulty of the exercises may be tailored to the individual’s abilities. In terms of efficacy, cognitive training has been demonstrated to enhance overall cognitive performance in persons with mild dementia [58]. Finally, cognitive rehabilitation is a tailored solution that is specifically tailored to a person’s needs [59]. Non-pharmacological therapies, such as cognitive interventions and exercise, may help to prevent and cure AD. Cognitive therapies have been shown to enhance cognition, prevent dementia, and increase cognitive reserve and plasticity [60].

Music therapy (MT) has been proven in several trials to improve multiple cognitive domains in AD patients, including attention, psychomotor speed, memory, orientation, and executive skills [61,62,63,64]. Moreover, according to Ozdemir L et al., the impact of MT on AD could persist for at least three weeks after the intervention [62].

Memory, orientation, depression, and anxiety (HAD scale) improved significantly in mild and moderate instances; anxiety (NPI scale) improved significantly in mild cases; and delirium, hallucinations, agitation, irritability, and language disturbances improved significantly in the moderate AD group. After only four sessions of music therapy, the effect on cognitive tests was noticeable [65]. De la Rubia Ort et al. assessed the effectiveness of a brief music therapy protocol as a treatment for stress reduction and emotional state improvement in individuals with moderate AD. The findings suggest that using this therapy reduces stress and greatly reduces sadness and anxiety, indicating a linear relationship between the variance of these variables and the variation of cortisol [66].

The study’s findings indicate that music therapy is beneficial for improving cognitive performance and mental well-being, and it may be suggested as an alternate strategy to control symptoms associated with AD [67]. Three different but interrelated processes can be proposed to explain the therapeutic effect of music on memory in AD. These include dopaminergic pathway activation, sympathetic excitation, and default neural connections [68].

### 2.5. Interventional Strategies

The parahippocampal gyrus-orbitofrontal cortex (PHG-OFC) circuit in humans is analogous to the postrural cortex (POR)-ventral lateral orbitofrontal cortex (vlOFC) circuit in rats. Both have been connected to visual impairment in AD. Zhu, L., and colleagues found that modification of the POR-vlOFC glutamatergic circuit alters visual memory in 5XFA cells. The PHG-OFC circuit explains the neuroanatomy of visuospatial memory problems in AD, providing a possible predictor of AD progression as well as a prospective therapeutic method for AD [69]

A study of the brain-penetrating microtubule stabilization drug epothilone D (EpoD) in aged PS19 mice with tau disease and related behavioral disorders. In aged PS19 mice, EpoD therapy decreased axonal degeneration and enhanced axonal microtubule density, resulting in better fast axonal transport and cognitive function [70]. In terms of reported outcomes, cognitive training had no positive or negative impact. The trials’ overall quality ranged from low to moderate. Cognitive rehabilitation training produced promising benefits on a variety of participant and caregiver outcomes and was typical of good quality [71].

Over the last two decades, research into the pathophysiology of AD has been primarily led by the amyloid hypothesis, with AD-associated neuroinflammation seen as a simple reaction to pathogenic events. However, emerging results from preclinical and clinical investigations show that immune-mediated responses contribute to the etiology of AD [72]. Cognitive training therapies, which have shown a synergistic impact with drugs, have emerged as complementary methods. Nonetheless, many cognitive therapies lack the intervention’s specificities, which is why their effectiveness is studied [73].

## 3. Effects of Natural Bioactive Compounds and Approved Drugs against AD

### 3.1. Phytochemicals against AD

Phytochemical investigations have demonstrated that bioactive molecules such as sterols, polyphenols, flavonoids, alkaloids, lignans, triterpenes, and tannins represent a major component of conventional prescription and have been extensively researched for their consideration in current medical exploration [6]. These secondary metabolites, abundant in diets, are commonly used as remedies for central nervous system diseases to improve human well-being and have been extensively screened for their AchE, Aβ and lipoxygenase inhibition activity, which may promote neuroplasticity and protect against neurodegeneration as well as neuroinflammation and oxidative stress in the brain, which leads to neuronal loss and thus promotes the development of AD [74]. In this sense, terpenes are among the main secondary metabolites providing neuroprotective activity. This has been well demonstrated in several investigations [75,76,77,78,79,80]. Xu et al. [77] reported that terpenes from *Alpinia oxyphylla* Miq exhibit neuroprotective activity on the formation and plasticity of dendritic spines and synapses in the nervous system and also control the synthesis, release, and transmission of neurotransmitters. Dash et al. [76] proved the anti-cholinesterase potential of penctylcurcumere terpene extracted from *G. repens*, the IC_50_ values of AchE and BchE were 73.12 ± 0.56 and 97.65 ± 0.46 μg/mL, respectively. Based on the investigation performed by Yılmaz et al. [75], the four terpenes isolated from *Nepeta obtusicrena* showed significant anti-Alzheimer’s effect, specifically against the AchE enzyme. Moreover, Suárez Montenegro et al. [78] assessed the terpenoids contained in olive leaf extract, which revealed a significant anti-cholinesterase effect in vitro without any toxicity at high concentrations. Romero Rocamora and colleagues [79] demonstrated a potent ability to suppress AchE in sage and rosemary essential oils, and screening of 16 terpenes by Shin et al. [80] revealed that the terpene limonene did not only affect the cellular level of Drosophila models of AD but also impaired neuroprotection, giving this terpene a potential preventive capacity and making it a promising component in the formulation of new therapies targeted against the AD model.

Alkaloids are a naturally occurring class of organic nitrogen compounds found primarily in plants, particularly in some flowering plant families such as *Ranunculaceae* (buttercups), *Papaveraceae* (poppies family), *Amaryllidaceae* (amaryllis), and *Solanaceae* (nightshades), which are particularly rich in alkaloids [81]. Numerous investigations have been directed toward elucidating the neuroprotective actions of natural alkaloids [81,82,83]. For example, galantamine obtained from plants belonging to the *Amaryllidaceae* family, used for many years in humans to manage neuropathic pain, is an alkaloid that may alleviate neurodegeneration in AD via neurogenesis and neuroprotection. Because it was found to suppress Aβ cytotoxicity and aggregation as well as stimulate adult neurogenesis in the mouse hippocampus through M1 muscarinic receptors and α7 nicotinic ACh receptors [84].

**Figure 1:**
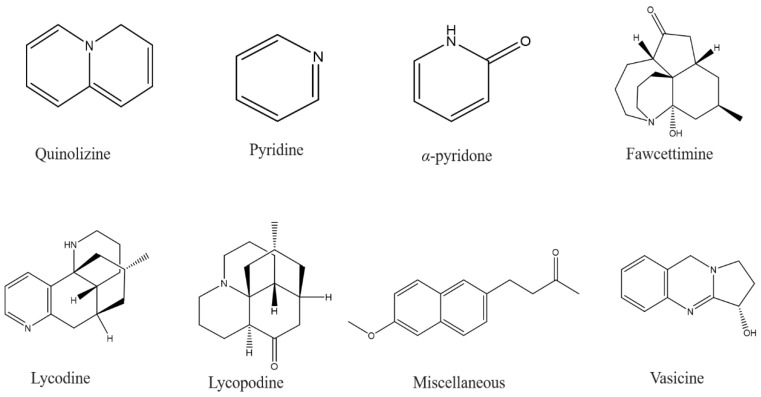
Structures of alkaloids with anti-Alzheimer effects.

The Lycopodium alkaloids are quinolizine, pyridine, or α-pyridone alkaloids (Figure 1), which can be classified into four distinct classes: fawcettimine, lycopodine, lycodine or flabellidine, and miscellaneous. The most important component of this group, huperzine A, is a lycodine-like alkaloid and was first extracted from the Chinese lycopod *Huperzia serrata* and was used to treat strains, fever, inflammation, swelling, and schizophrenia for centuries. It showed neuroprotective activity against neuronal apoptosis in AD, which can be mediated through the regulation of pro-apoptotic protein expression and enhanced expression of anti-apoptotic genes/proteins [81]. Moreover, some indole alkaloids, such as angustine, vallesiachotamine, and deoxyvobtusine, are more selective for BchE. [85]. Juliflorine is a piperidinium alkaloid extracted from *Prosopis juliflora* leaves and consists of two piperidines linked to a dihydroindilizine moiety by two aliphatic chains. This compound inhibited AchE and BchE in a dose-dependent manner with an IC_50_ of 0.42 and 0.12 μm, respectively [86]. Using the Thai marine sponge *Petrosia* n. sp., the alkaloid pyridoacridine petrosamine was obtained. This compound occurs in both enolate and ketone forms and exhibits a strong inhibitory pattern for AchE, with an IC_50_ of 0.091 μm [87]. Brunhofer et al. determined three quinazoline alkaloid compounds that could be used as leads to target key mechanisms involved in the pathogenesis of AD. The findings indicated that these compounds are selective BchE inhibitors. Among them, vasicine was similarly potent in inhibiting eqBchE (IC_50_ = 2.53 μm) and hBchE (IC_50_ = 3.13 μm). Furthermore, deoxypeganin was a non-selective inhibitor. It is equipotent for eqBchE (IC_50_ = 11.8 μm) and eqAchE (IC_50_ = 11.9 μm), and finally peganole seems more selective for eqBchE (IC_50_ = 11.4 μm) than for hBchE (IC_50_ > 20 μm) [88].

**Figure 2:**
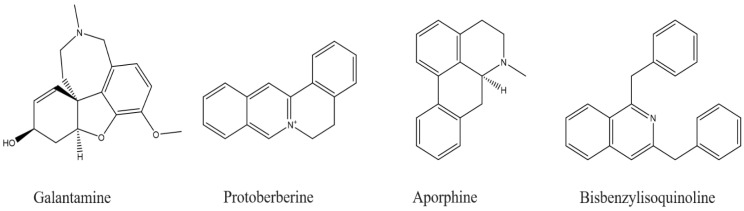
Structures of isoquinolines with anti-Alzheimer effects.

On the other hand, the phytochemical profile of *Enhydra fluctuans*, a favorite vegetable in Bangladesh used in folk medicine to cure nervous system diseases, showed potently beneficial effects against AD due to its secondary metabolite content, including tannins, phenolics, flavonoids, alkaloids, saponins, and phytosterols. It was found that *E. fluctuans* chloroform extract contained 41.84 mg catechin equivalent/g extract of flavonoids and 19.16 mg gallic acid equivalent/g extract of phenolics. Furthermore, *E. fluctuans* chloroform extract was found to exhibit the strongest inhibition against both butyrylcholinesterase (IC_50_ = 48.14 μg/mL) and acetylcholinesterase (IC_50_ = 83.90 μg/mL) enzymes, as evidenced by the spectrophotometric method [89]. The main classes of compounds possessing important anti-enzymatic properties in the prevention and protection against AD are the isoquinolines (especially the galantamine, protoberberine, aporphine, and bisbenzylisoquinoline classes) (Figure 2), the steroids/triterpenoids (most of which selectively inhibit BchE), the quinolizidines (especially huperzine A), and the indole alkaloids. They are still relatively marginal and require further study to know precisely the action mechanisms and targets of these terpenes in order to design effective therapies.

### 3.2. Current Approved Drugs in AD Treatment

New AD drugs are needed, but multiple clinical trials of experimental drugs have yielded no promising options. Recently, Rodriguez S. et al. developed a novel artificial intelligence-based approach, Drug Repurposing in AD (DRIAD), which involves a machine learning framework that quantifies the association between any biological process or response that can be characterized by a list of gene names and the stage of AD (early, middle, or advanced) as defined by Braak’s staging [90].

Additionally, Jang et al. [91] demonstrated an efficient network-based drug screening platform designed by integrating mathematical modeling and pathological features of AD with iPSC-derived human brain organoids (iCOs), including CRISPR-Cas9-edited isogenic lines [91]. In contrast, PTI-125 is a small-molecule oral drug candidate that binds and reverses the altered conformation of filamin A, a scaffolding protein found in the brains of patients with AD. Given the promising biomarker data and safety profile, about 60 patients with mild-to-moderate AD are currently being recruited into a randomized, placebo-controlled, confirmatory Phase 2b study to assess the tolerability, efficacy, and safety of ITP-125 [92]. Usually, the United States (U.S.) FDA-approved medication for AD includes AchE inhibitors and N-methyl-D-aspartate receptor (NMDAR) antagonists.

#### 3.2.1. Acetylcholinesterase Inhibitors

Currently, since acetylcholine levels and neurotransmitter destabilization gradually decline in AD, which seems to be related to altered memory and cognition, cholinesterase inhibitors that raise endogenous acetylcholine content have been adopted as key strategic drug therapies for the management of cognitive and behavioral symptoms in mild to moderate forms of AD [93]. The three AchE inhibitors most commonly prescribed are donepezil (Aricept^®^: approved to treat all stages of AD), rivastigmine (Exelon^®^: approved for mild-to-moderate Alzheimer’s as well as mild-to-moderate dementia associated with Parkinson’s disease), and galantamine (Razadyne^®^ or Reminyl^®^: approved for mild-to-moderate stages of AD), which were approved in 1996, 2000, and 2001, respectively [94]. Donepezil is highly selective for AchE over BchE. Galantamine and donepezil are AchE inhibitors, whereas rivastigmine is a reversible inhibitor of both AchE and BchE [95]. Tacrine provided cognitive benefits in 5–40% of patients with mild-to-moderate AD after therapy and was the first drug approved by the FDA and introduced to the market for the treatment of AD. However, gastrointestinal side effects and severe dose-related hepatotoxicity have been noted [82]. In addition, physostigmine derivatives, such as phenserine, tolserine, and eseroline, have been developed as next-generation of AchE inhibitors. Phenserine was observed to be a promising agent for the development of new AD treatment strategies due to its dual anti-Aβ and anti-AchE effects [96]. In 2000, preclinical studies concluded that tolserine is 200-fold more selective against human AchE (hAchE) than BchE. The inhibitory concentration of tolserine against AchE in human erythrocytes is 0.01 µM [97]. Eseroline has been shown to be a metabolite of physostigmine, but unlike physostigmine, the effect of eseroline on AchE inhibition is both limited and reversible [95,98]. Because AD is a multifactorial disorder, dual hybrids, synthetic analogs, and multi-target inhibitors have been designed. For example, donepezil-AP2238 hybrid, Donepezil-tacrine hybrid, Tacrine-ferulic acid (T6FA) hybrid, Tacrine, and 8-hydroxyquinoline hybrids are forming the hybrid cholinesterase inhibitors. At the same time, heteroarylacrylonitrile derivative, tacrine analog, Ladostigil, and indenyl derivative are considered synthetic analogs of cholinesterase inhibitors [95]. The use of cholinesterase inhibitors appears to have a beneficial impact on behavioral and psychiatric symptoms, but there is no evidence of the superiority of one agent over another with respect to cognitive, behavioral, or functional outcomes.

#### 3.2.2. N-methyl-D-aspartate Receptor Antagonist

N-methyl-D-aspartate receptor (NMDAR) is thought to play a pivotal role in the pathophysiology of AD. Stimulation of NMDAR causes an influx of Ca^2+^ which activates signal transduction and thereby triggers the transcription of genes essential for the formation of long-term potentiation (LTP), which is critical for memory formation, plasticity, and synaptic neurotransmission [99]. Since its approval by the FDA in 2003, memantine (Namenda™) has emerged as the first revolutionary drug for the management of patients with moderate to severe AD and offers a novel mechanism of action (non-competitive, a moderate affinity, voltage-dependent NMDA receptor antagonist with rapid on/off kinetics) to regulate glutamatergic failure. It improved patients’ performance related to cognition, behavioral problems, and daily functioning. Memantine is a unique non-competitive NMDA antagonist that certainly ensures its position as a classic neurochemical that can be used alone or in combination with other AChE inhibitors, such as donepezil [100,101]. Additionally, other non-competitive NMDAR antagonist compounds are under investigation, such as RL-208 (3,4,8,9-tetramethyltetracyclo [4.4.0.0^3,9^.0^4,8^]dec-1-yl)methylamine hydrochloride), a polycyclic amine compound that could have a potentially beneficial therapeutic outcome in age-related cognitive impairment and AD [101].

## 4. Mechanistic Pathways (Anti-Inflammatory, Antioxidant, Anti-Amyloidogenic, Anti-Autophagy) of Phytochemicals Involved in AD Progression Blockage

Previous investigations have indicated that natural bioactive molecules from phytochemicals exhibit anti-inflammatory, anti-amyloidogenic, and antioxidant activities as well as targeting autophagy; thus, they may attenuate the progression of AD damage. Many polyphenols, sterols, alkaloids, flavonoids, and vitamins have shown significant ability to prevent neurodegenerative diseases and may be interesting drug candidates for the treatment of AD [12,13,102]. In this sense, a polyphenolic compound as resveratrol present in seeds, red wines, and grape skins, has anti-inflammatory and antioxidant characteristics; the beneficial effects of this compound in AD have been reported in in vitro and in vivo models, as it maintains metal homeostasis and increases mitochondrial function [103]. The in vitro study conducted by Feng X et al. used PC12 cells to assess the role of resveratrol in protecting against β-amyloid peptide 25-35 (Aβ(25-35)) neurotoxicity. The results showed that resveratrol protected P12 cells against Aβ-induced cell apoptosis, alleviated Aβ(25-35) neurotoxicity, stabilized intercellular Ca^2+^ homeostasis via upregulation of the silent information regulator 1 (SIRT1), and downregulation of rho-associated kinase 1 (ROCK1) by SIRT1. Hence, the SIRT1-ROCK1 pathway may be critical in the pathophysiology of AD [104]. Furthermore, in the in vivo experiment using SAMP8, dietary resveratrol (1 g/kg in the diet/7 months) has a marked neuroprotective effect, increased life expectancy, reduced cognitive impairment, as well as decreased amyloid load and tau hyperphosphorylation. Additionally, they found that resveratrol supplementation leads to the activation of AMPK and SIRT1 pathways as well as the non-amyloidogenic ADAM-10 enzyme [105]. An isoflavone widely present in certain plants such as soybeans, chickpeas, and fava beans, genistein might play a crucial role in the treatment of AD, as evidenced by various investigations. In the in vitro model, Zhou, X et al. showed that genistein treatment induces increased cell viability and attenuates Aβ25–35-induced inflammatory damage in BV-2 microglia cells via the Toll-like receptor 4 (TLR4)/NF-κB signal pathway [106]. In addition, it was found that the combinations of galantamine and genistein exhibit high neuroprotective activity by reducing β-amyloid peptide (Aβ(1-42)) induced cell death and genotoxicity in the SH-SY5Y cell line in vitro [107]. Similarly, some recent in vivo research has indicated that genistein, as one of the selective estrogen receptor modulators (SERMs), can enhance brain function across the blood–brain barrier (BBB), suppress neurotoxicity caused by beta-amyloid protein aggregation, and exhibit neuroprotective benefits [108].

The polyphenol derived essentially from green tea, (-)-epi-gallocatechin gallate (EGCG) (15 and 20 μM for one to three days), was able to convert large and mature amyloid-β fibrils into smaller, amorphous, and nontoxic aggregates in HEK-293 cells in vitro. This bioactive molecule could bind directly to *β*-sheet-rich aggregates and mediate conformational change [109]. In the in vivo study, chronic dietary supplementations of EGCG (50 mg/kg for six months) in AD transgenic mice enhanced the nonamyloidogenic processing of APP and decreased Aβ levels, tau and plaques by up-regulating α-secretase activity [110].

On the other hand, a flavonoid present in various food sources such as broccoli, red onions, apples, and tea, quercetin promotes antioxidant enzyme function and glutathione (GSH) levels and possesses antioxidant, anti-inflammatory, and anti-apoptotic activities [111]. Significant interest has been directed toward the increase in intracellular GSH levels in some diseases, particularly AD. In the in vitro investigation, Ansari et al. [112] showed that quercetin at low pretreatment doses significantly improved Aβ(1-42) induced lipid peroxidation, protein oxidation, apoptosis, and cytotoxicity in cultured neurons but was toxic and not neuroprotective at high doses [112]. Sabogal-Guáqueta et al. performed a study to evaluate the in vivo neuroprotective effect of quercetin treatment (25 mg/kg, every 48 h for three months) in aged triple transgenic mice of the AD model (3xTg-AD). The results demonstrated that quercetin improves learning and memory function by decreasing Aβ1-40, Aβ1-42, and BACE1 levels and the paired helical filament (PHF) as well as reducing microglial activation [113]. The 10 mg/kg dose of quercetin administered to rats injected with Aβ1-40 intrahippocampally was found to improve learning and memory and short-term spatial recognition memory in a Y-maze test. Moreover, this biomolecule markedly attenuated the increase in MDA levels without affecting nitrite levels or SOD activity. Therefore, it attenuated oxidative stress [114]. Furthermore, quercetin supplementation in APPsw/PS1dE9 transgenic mice ameliorated memory and learning deficits and plaque burden. It seems that activation of AMPK promotes the protective effects of quercetin while reducing mitochondrial dysfunction [115]. In addition, quercetin was able to reduce pro-inflammatory mediators such as iNOS, IL-1β, and COX-2, as well as decrease β-amyloid plaque aggregation in the CA1 hippocampal region of aged triple transgenic mice with AD [116].

Curcumin is an active polyphenolic ingredient derived from dried turmeric roots. In an in vivo model, curcumin improves cognitive function, decreases α-synuclein A53T accumulation, and inhibits Aβ aggregation in curcumin-induced autophagy in APP/PS1 double transgenic mice through down-regulation of the PI3K/Akt/mTOR pathway, which is suggested to be the mechanism responsible for autophagy stimulation [117]. However, in an in vitro study, this polyphenolic compound was found to exhibit neuroprotective effects by enhancing the expression of antioxidant and anti-apoptotic genes. It alleviates paraquat-induced cell death in SH-SY5Y cells by modulating autophagy and oxidative stress [118]. It was also reported by Huang et al. [119] that tau hyperphosphorylation induced by Aβ in human neuroblastoma SH-SYSY cells was suppressed by curcumin, and this was associated with the PTEN/Akt/GSK3β signaling pathway [119].

Hesperidin or hesperetin, a glycoside flavonoid found in citrus fruits such as oranges and lemons, may represent a promising agent in preventing the progression of AD. The intragastric administration of hesperidin in APP/PS1 mice (40 mg/kg for 90) improved learning and memory defects by attenuating inflammation and oxidative stress via activation of Akt/Nrf2 signaling and inhibition of RAGE/NF-κB signaling [120]. Furthermore, using APP/PS1-21 transgenic mice, treatment with hesperidin markedly recovered deficits in both nesting and social interactions and alleviated microglial activation, Aβ deposition, and levels of pro-inflammatory mediators such as TNF-α, IL-1β, and iNOS in transgenic APP/PS1 mice [121]. The administration of hesperidin was reported by Huang et al. [122] to enhance Aβ1-42 impaired glucose utilization, particularly by reducing Aβ-induced cellular autophagy in Neuro-2A cells [122].

As an example of a class of triterpene saponins, the bioactive compound ginsenoside-Rg2, extracted from *Panax ginseng*, triggers autophagy in an mTOR-independent manner and ULK1/AMPK-dependent manner. The natural drug Rg2 enhanced aggregate protein clearance and significantly enhanced cognitive performance in an AD mouse model by promoting autophagy [123].

On the other hand, interest has also turned to lycopene as an agent with potential utility in the treatment of AD. The anti-inflammatory, anti-apoptotic, and antioxidant effects of lycopene may be related to its neuroprotective property [124]. The in vitro study performed by Qu M. et al. [125] demonstrated that lycopene pretreatment significantly alleviated Aβ(25-35)-induced neurotoxicity in cultured rat cortical neurons, as evidenced by the decreased apoptosis rates and improved cell viability levels. Furthermore, lycopene suppressed Aβ(25-35)-induced ROS production and mitochondrial membrane potential depolarization. Lycopene also blocked caspase-3 activation and restored anti-apoptotic Bcl-2 and pro-apoptotic Bax levels.

The majority of medicated feed homology varieties have been shown to exhibit neuroprotective benefits and ameliorate brain deficits through inhibition of neuropathological hallmarks (amyloid precursor protein, Aβ-induced toxicity, and phosphorylated tau immunoreactivity), including antioxidant stress, anti-inflammation, anti-apoptosis, and anti-autophagy. Consequently, they may provide important benefits for individuals’ healthy lifestyles and the development of drugs for the treatment and prevention of AD [126].

## 5. Nanoformulations and Nanoparticles as Phytochemical Delivery Systems against AD

Many natural compounds demonstrate low levels of solubility, stability, bioavailability, and target specificity in the body, which makes it unrealistic for these compounds to be present at their effective levels in the target tissues [14]. In this context, the nanoformulation of these compounds into nanoparticles opens up a new way to overcome the aforementioned problems. Phytochemicals can be encapsulated into biocompatible and biodegradable nanoparticles to improve their bioavailability, aqueous solubility, and stability [15]. The best types of nanoparticles used for drug delivery and the biodistribution of phytochemicals and natural products include polymeric nanoparticles, nanoliposomes, and nanoemulsions [16].

Liposomes are nano-sized spherical vesicles consisting of one or more concentric lipid bilayers that encapsulate a small amount of the solvent in which they freely diffuse. Most often, liposomes are composed of phospholipids, such as phosphatidylcholine, and are synthesized by several methods, such as extrusion, sonication, micro-emulsification, and microfluidization [127]. Due to their biphasic character, liposomes can serve as carriers for both hydrophilic phytochemicals such as EGCG that can be encapsulated into the hydrophilic core of liposomes and hydrophobic compounds such as resveratrol, curcumin, and quercetin that can be incorporated into the hydrophobic domain of their phospholipid tails [128], which increased their stability, aqueous solubility, and bioavailability and improved their biological activities [129]. Indeed, Moballegh-Nasery et al. (2021) [130] reported that the cytotoxic activity of curcumin against breast cancerous cell lines (MCF-7 and SKBR3 cells) was improved by its encapsulation in both liposomes and affibody-decorated liposomes. In addition, encapsulation in liposomes with a combination of curcumin and resveratrol significantly decreases prostatic adenocarcinoma growth through the activation of apoptosis and modulation of p-Akt, cyclin D1, mTOR, and androgen receptor [131]. Yekollu et al. showed that curcumin-containing liposomes improved insulin resistance in the leptin-deficient and reduced blood fasting glucose and insulin levels, as well as decreased inflammatory responses in obese mice models of insulin resistance [132]. On the other hand, developed liposomal formulations significantly reduced elevated glucose levels in diabetic cell groups synchronous with increasing insulin levels and showed prolonged antioxidant activity against oxidative stress compared to RSV solution [133]. In another study, the administration of quercetin-loaded liposomes attenuated bleomycin-induced pulmonary fibrosis in vivo by suppressing inflammatory cytokines, including TNF-α, IL-1β, and IL-6 [134]. Khan et al. demonstrated better efficacy of liposomal formulation of thymoquinone, which significantly reduced the level of blood glucose and hepatic inflammation markers and repaired kidney functioning in diabetic mice [135]. In diabetic cardiomyopathy rats, the administration of Apigenin-loaded nanosized liposomes alleviated hyperglycemia and inhibited apoptosis of myocardial cells via the Bcl-2/Bax pathway compared with Apigenin [136]. The in vitro study performed by Shu et al. [137] demonstrated that betulinic acid-loaded liposomes pretreatment had exhibited excellent anticancer activity against HepG2 cells by inhibiting the proliferation and promoted cell apoptosis and strengthening the destruction of mitochondrial membrane potential in HepG2 cells.

Nanoemulsion is a mixture composed of two immiscible liquids (aqueous and oil) and an emulsifier with droplet sizes ranging from 50–1000 nm. These nanoemulsions are synthesized by micro-fluidization and high-pressure homogenization techniques [138]. Various works have been conducted to use nanoemulsions to encapsulate bioactive compounds and drugs due to their favorable properties such as biocompatibility, bioavailability, large surface area, improved solubility of lipophilic drugs, and their presence suiting absorption at the gastric and intestinal levels [129]. Several phytoconstituents have been formulated into nanoemulsion form for the treatment of numerous diseases. Young and collaborators [139] reported that encapsulation of curcumin by nanoemulsions increases their bioavailability and suppresses inflammatory-induced NF-κB signaling and macrophage migration in mice. Moreover, in experimental diabetic neuropathy, self-nano-emulsified curcumin formulation improved its bioavailability and protection against pain and functional deficits [140]. The nanoemulsified resveratrol also better preserved the antioxidant activity of resveratrol during the digestion process and increased both their stability and bioavailability [141]. Furthermore, encapsulation of carvacrol, limonene, cinnamaldehyde, and thymol into nanoemulsions enhances their antimicrobial properties against several bacterial strains and improves their anticancer and antioxidant activities [142].

Polymeric (NPs) consisting of a reservoir system (nanocapsule) and matrix system (nanosphere) that can be synthesized from the polymer of natural (gelatin, pectin, sodium alginate) or synthetic origin (PLGA, PBA, PLA) using emulsification–diffusion, nanoprecipitation, and solvent evaporation methods [143]. The polymeric materials encapsulate phytochemical and protect from external degradation with enhanced stability for long-term storage as well as increased bioavailability and improved sustained and controlled release manner. Resveratrol-loaded PLGA nanoparticles improved the stability, solubility, absorption, bioavailability, and sustained release of resveratrol to attenuate non-alcoholic fatty liver disease associated with type 2 diabetes [144]. Nanoformulated resveratrol using nano-particles based on poly(epsiloncaprolactone) and poly(D,L-lactic-co-glycolic acid)-poly(ethylene glycol) also demonstrated anticancer activities against human prostate cancer DU-145, PC-3, and LNCaP cell lines by decreasing cell growth and proliferation [145]. Samadder et al. [146] reported that LGA nanoparticle encapsulation of pelargonidin attenuates hyperglycemia-triggered apoptosis, oxidative stress, DNA damage, and impaired glucose utilization compared to native pelargonidin. Moreover, formulated curcumin-loaded PLGA nanoparticles increased anticancer activity against prostate cancer [147]. In another study, cinnamaldehyde and eugenol were loaded into PLGA nanoparticles to enhance their controlled release and antimicrobial activities against several bacterial stains [148].

On the other hand, several findings of neurodegenerative disorders revealed the potential therapeutic effect of nanocurcumin in this pathology by regulating multiple pathways. Fan et al. [149] reported that curcumin-loaded poly(lactide-co-glycolide)-block-poly(ethylene glycol) (PLGA-PEG) nanoparticles conjugated with B6 peptide (PLGA-PEG-B6/Cur) enhance learning spatial and memory capability of APP/PS1 transgenic mice. Using Bielschowsky silver staining, immunostaining, and Western blotting, the authors demonstrated that this nanoparticle reduces hippocampal β-amyloid formation and deposition and tau hyperphosphorylation. Likewise, Brahmkhatri et al. [150] showed that the polymeric nanoparticle-encapsulated curcumin conjugate with gold nanoparticles decorated on the surface (PVP–C–AuNP) inhibits the aggregation of N-terminal region (Ab-16) of a beta-amyloid peptide that characterized AD. In another study, curcumin-encapsulated biodegradable poly(lactic-co-glycolic acid) (PLGA) nanoparticles (Cur-PLGA-NPs) potently induced adult neurogenesis and reversed cognitive deficits in the AD model by internalization into the hippocampal NSC, increasing expression of genes involved in cell proliferation and neuronal differentiation and increasing neuronal differentiation through activation of the canonical Wnt/β-catenin pathway [151]. Encapsulation of curcumin in a biodegradable poly (lactide-co-glycolide) (PLGA) based-nanoparticulate formulation (Nps-Cur) also showed neuroprotective activity against neuronal oxidative damage by decreasing ROS, accompanied by increased glutathione (GSH) concentrations and the oxidized glutathione (GSH: GSSH) ratio in human neuroblastoma (SK-N-SH) cells. It also suppressed Nrf2 activation [152] and decreased caspase-3 and caspase-7 activities in the brain [153]. Moreover, the PLGA-curcumin nanoparticles are able to disaggregate the amyloid proteins slowly. It was observed that these nanoparticles were able to attach to the surface of amyloid aggregates and decrease the size of the aggregates [154]. Moreover, the administration of curcumin-loaded lipid-core nanocapsules (CurLNC) at a 20-fold dose in an animal model of AD significantly improved cognitive impairment induced by i.c.v. Ab injection by a significant decrease in GFAP levels and an increase in brain-derived neurotrophic factor expression, Akt phosphorylation levels, and GSK-3b phosphorylation/inactivation. It also significantly attenuated increased levels of TNF-α and IL-1β [155]. Using surface plasmon resonance experiments, the nanosized curcumin-decorated liposomes showed the highest affinity for Aβ1-42 fibrils (1–5 nM), to be used as vectors for targeted delivery of new diagnostic and therapeutic molecules for AD [156]. Resveratrol is a natural stilbene-class of polyphenol compound and has attracted remarkable attention for its anti-inflammatory and neuroprotective activity [157]. In aluminum chloride-induced AD model mice, resveratrol-selenium nanoparticles (RSV-SeNPs) alleviate neurotoxicity by up-regulating sirtuin-1 (SIRT1) expression and down-regulating that of microRNA-134, which increases neurite outgrowth [158]. Additionally, RSV-SeNPs inhibited Aβ Aggregation, decreased ROS, and increased antioxidant enzyme activity [159] as well as down-regulated neuroinflammation via the NF-κB/mitogen-activated protein kinase (MAPKs)/Akt signal pathway [160]. In another study, treatment with resveratrol-loaded polymeric micelles protected PC12 cells from Aβ-induced damage-inhibited cells via attenuation of oxidative stress and caspase-3 activity [161]. Furthermore, Frozza and collaborators [162] reported that treatment with a resveratrol-loaded lipid-core nanocapsule protects organotypic hippocampal cultures against inflammation and cell death induced by Aβ1-42 by increasing IL-10 and by decreasing both glial and JNK activation and ROS formation. Indeed, the treatment of PC12 cells with EGCG-stabilized selenium nanoparticles coated with Tet-1 peptide was reported by Zhang et al. [163] to inhibit Aβ fibrillation and disaggregate preformed Aβ fibrils into nontoxic aggregates. The inhibition of Al(III)-induced Aβ-42 fibrillation and its neurotoxicity was also reported in another study using nano-EGCG [164]. Likewise, oral treatment of APPswe/PS1dE9 (APP/PS1) mice, as AD model mice with epigallocatechin-3-gallate nanoparticles, significantly improved spatial learning and memory by decreasing neuroinflammation, amyloid β (Aβ) plaque burden, and cortical levels of soluble and insoluble Aβ(1-42) peptide [165]. Quercetin is a widely distributed flavonoid compound in plants. Several studies reported that quercetin nanoparticles (NQC) enhanced its bioavailability and showed effective neuroprotective action in cellular and animal models of AD. The in vivo study performed by Rifaai et al. [166] reported that quercetin nanoparticles attenuate AlCl3-induced neurotoxicity in mice via several mechanisms. It decreased neuronal degenerative changes, amyloid plaques and NFT formation, restored TH activity, and enhanced regenerative changes. Treatment of the SAMP8 mouse model of AD with nanoencapsulated quercetin improved cognition and memory impairments and reduced expression of the hippocampal astrocytic marker GFAP [167]. Furthermore, the injection of PLGA-quercetin nanoparticles into APP/PS1 mice ameliorated cognition and memory impairment [168]. In another study, Quercetin-modified gold-palladium nanoparticles protected SH-SY5Y cells from Aβ-induced cytotoxicity damage as well as promoted degradation of autophagosomes and lysosomes and accelerated the clearance of Aβ [169]. Other nanoformulations that have been shown to trigger similar effects in AD are ferulic acid-loaded lipid nanostructures [170,171], quinoline-n-butylcyanoacrylate-based nanoparticles [172], naringenin nanoemulsion [173], sialic acid-conjugated dendrimers [174], and Trimethylated chitosan-conjugated PLGA nanoparticles [175]. Mechanistically, these nanophytochemicals can effectively cross the BBB, reduce ROS generation, increase neurogenesis, and attenuate Aβ-induced neurotoxicity.

## 6. Conclusions and Future Perspectives

Here, we present the risk factors, the pathophysiology as well as the major therapeutic approaches used for the treatment of this pathology. It was shown that different therapeutic strategies are used to fight against Alzheimer’s disease, including the use of natural drugs. Indeed, these bioactive compounds have shown significant anti-Alzheimer effects with different cellular and molecular mechanisms. However, some actions, including the effects of natural compounds and epigenetic pathways involved in this pathology, have not yet been understood, and therefore, further studies are needed to further investigate this aspect. On the other hand, the effects of these molecules are also enhanced by nanoformulations. However, the nanoparticles used are limited, and other studies should be carried out to develop other nanoparticles with the ability to improve the efficacy of phytochemical compounds against Alzheimer’s disease.

## Data Availability

Not applicable.
